# Feasibility of late acquisition [68Ga]Ga-PSMA-11 PET/CT using a long axial field-of-view PET/CT scanner for the diagnosis of recurrent prostate cancer—first clinical experiences

**DOI:** 10.1007/s00259-021-05438-5

**Published:** 2021-06-21

**Authors:** Ian Alberts, George Prenosil, Clemens Mingels, Karl Peter Bohn, Marco Viscione, Hasan Sari, Axel Rominger, Ali Afshar-Oromieh

**Affiliations:** 1 Department of Nuclear Medicine, Inselspital, Bern University Hospital, University of Bern, Freiburgstr. 18, 3010 Bern, Switzerland; 2 Advanced Clinical Imaging Technology, Siemens Healthcare AG, Lausanne, Switzerland

**Keywords:** Total body, Ultra-long FOV PET, Whole body, PET/CT, Positron-emission tomography, Digital PET

## Abstract

**Purpose:**

While acquisition of images in [^68^ Ga]Ga-PSMA-11 following longer uptake times can improve lesion uptake and contrast, resultant imaging quality and count statistics are limited by the isotope’s half-life (68 min). Here, we present a series of cases demonstrating that when performed using a long axial field-of-view (LAFOV) PET/CT system, late imaging is feasible and can even provide improved image quality compared to regular acquisitions.

**Methods:**

In this retrospective case series, we report our initial experiences with 10 patients who underwent standard imaging at 1 h p.i. following administration of 192 ± 36 MBq [^68^ Ga]Ga-PSMA-11 with additional late imaging performed at 4 h p.i. Images were acquired in a single bed position for 6 min at 1 h p.i. and 16 min p.i. at 4 h p.i. using a LAFOV scanner (106 cm axial FOV). Two experienced nuclear medicine physicians reviewed all scans in consensus and evaluated overall image quality (5-point Likert scale), lesion uptake in terms of standardised uptake values (SUV), tumour to background ratio (TBR) and target-lesion signal to background noise (SNR).

**Results:**

Subjective image quality as rated on a 5-point Likert scale was only modestly lower for late acquisitions (4.2/5 at 4 h p.i.; 5/5 1 h p.i.), TBR was significantly improved (4 h: 3.41 vs 1 h: 1.93, *p* < 0.001) and SNR was improved with borderline significance (4 h: 33.02 vs 1 h: 24.80, *p* = 0.062) at later imaging. Images were obtained with total acquisition times comparable to routine examinations on standard axial FOV scanners.

**Conclusion:**

Late acquisition in tandem with a LAFOV PET/CT resulted in improvements in TBR and SNR and was associated with only modest impairment in subjective visual imaging quality. These data show that later acquisition times for [^68^ Ga]Ga-PSMA-11 may be preferable when performed on LAFOV systems.

**Supplementary Information:**

The online version contains supplementary material available at 10.1007/s00259-021-05438-5.

## Introduction

Recently introduced long axial field-of-view (LAFOV) PET/CT scanners offer significant improvements over previous standard axial FOV (SAFOV) systems [1, 2], with substantially improved sensitivity and count densities [3]. Most recently, the first Siemens Biograph Vision Quadra system (Siemens Healthineers, Knoxville, TN, USA) with an axial FOV of 106 cm was installed at the Department for Nuclear Medicine, University Hospital Bern, in Switzerland. The performance characteristics of this scanner have been investigated previously in phantom studies [4, 5] and in a clinical setting [6].

PET/CT with PSMA radioligands is the modality of choice for the staging of biochemically recurrent prostate cancer (PC). Typically, when performed with [^68^ Ga]Ga-PSMA-11, imaging is routinely performed at 1 h post injection of radiotracer (p.i.) [7–9]. However, this choice of imaging time is drawn from the first clinical description of [^68^ Ga]Ga-PSMA-11, and is not based on a systematic evaluation of the optimal imaging time [7, 10]. Acquisition of images at later time points is associated with improved tumour to background (TBR) and lesion detectability [11–15] and is mentioned in current guidelines [9]. Despite these benefits to later acquisition, limiting factors are encountered: e.g. the short half-life of the radiopharmaceutical (68 min) results in decay at later imaging, and additional scanning can be difficult to integrate into a busy clinical service. This is particularly the case where, as a result of radioisotope decay, longer acquisition times are required to achieve adequate count statistics which can reduce the scanner’s availability for other patients [9]. Pioneering publications with LAFOV scanners report increased dynamic range for LAFOV systems, meaning that radiopharmaceuticals can be followed usefully over more half-lives as a result of the scanner’s higher sensitivity [1]. The aim of this short communication is to report our initial experiences with a LAFOV scanner in late imaging with [^68^ Ga]Ga-PSMA-11, to demonstrate that later acquisition of imaging is feasible and that even improved image quality can be obtained at late imaging when compared to regular acquisitions.

## Materials and methods

### Study design

This retrospective case series presents 10 cases who underwent “standard” clinical [^68^ Ga]Ga-PSMA-11 PET/CT on our LAFOV system for biochemically recurrent PC at 1 h p.i. and where additional “late” imaging was performed as per institutional standard. Previously, such additional scans were performed at 2.5 h p.i. using a SAFOV-PET [13]. Cognisant of the higher sensitivity of the LAFOV system, additional late imaging for these patients was performed at 4 h p.i.

### Imaging procedures

All scans were performed using the Siemens Biograph Vision Quadra system (axial FOV = 106 cm). Reconstruction parameters were as previously described [13]. Patients underwent a single scan from head to mid thighs in a single bed position (b.p.) with acquisition times of 6 min at 1 h p.i. and 16 min at 4 h p.i. (including an additional low dose CT for attenuation correction) following a single bolus administration of the radiopharmaceutical (192 ± 36 MBq) which was prepared as previously published [16]. Thirty minutes prior to the late (4 h) scans, 20 mg of intravenous Furosemide was applied following oral hydration in compliance with local protocol [13].

### Image analysis

All scans were analysed in a consensus read by two experienced nuclear medicine physicians (first and last author). Scans were analysed first with respect to target lesions according to published interpretation criteria [17]. PET images were displayed in the PET-rainbow colour look-up table using appropriate software (Syngo.Via, Siemens Healthineers) as previously described [18]. Both readers were blinded to both patient demographics and image acquisition details. To minimise recall bias, images were reviewed in randomised order. Images were rated by both readers according to subjective image quality rated on a 5-point Likert scale: (1) very poor; (2) poor; (3) acceptable; (4) very good; (5) excellent. Lesion uptake was measured in terms of SUV_peak_ [19], which has been shown to be a more reliable parameter irrespective of acquisition time than SUV_max_ [20]. The background was defined as the SUV_mean_ of a background volume of interest (VOI) placed in a reference region of healthy liver tissue, where SUV_mean_ was shown to be the most reliable parameter for liver [11, 19]. TBR was defined as lesion SUV_peak_ divided by background SUV_mean_. SNR was defined as the reciprocal coefficient of variation SNR = $\mu /\sigma$, where μ = target lesion SUV_peak_ and σ = standard deviation of the liver-background VOI [21]. The data in this case series were analysed descriptively using mean, median and statistical significance testing by means of the paired Student’s *t*-test.

## Results

Patient characteristics are shown in Table 1. All patients have PSMA-avid prostate cancer lesions, with a total of 20 pathological lesions detected and analysed. As anticipated, subjective image quality (as rated on a Likert scale) was judged to be highest for the acquisitions at 1 h p.i. (mean 5/5), with only modest reduction in visual quality observed at 4 h p.i (mean 4.2/5, range 4–5) as rated on a 5-point Likert scale.

**Table 1:** Patient characteristics: *RPE* radical prostatectomy, *RT* radiotherapy, *ADT* androgen deprivation therapy, *None* no further treatment, *Scan (p.i.)* scan time post injection of radiotracer hh:mm. Scan findings: *LR* local recurrence, *LN* lymph node, *X* no PSMA-avid lesions suspicious for PC, *pulm* pulmonary metastasis

Patient	Age (years)	Body weight (kg)	Applied activity (MBq)	PSA (ng/ml)	Gleason score	Initial therapy	Further therapy	Scan 1 (p.i.)	Scan 2 (p.i.)	Scan findings
1	78	60	217	1.5	9	RPE	ADT	01:05	03:55	LR
2	74	85	210	2.11	8	RPE	RT	01:10	04:08	LR, pelvic LN
3	67	62	146	2.2	7	RPE	RT + ADT	01:03	04:13	LR
4	78	102	211	2.15	7	RT	ADT	01:24	04:25	LR, bone
5	60	85	161	4.52	7	RPE + RT	ADT	01:04	04:02	bone
6	63	60	180	0.44	7	RPE	None	01:01	04:11	LR, pelvic LN
7	75	75	185	5.2	7	RPE	None	01:16	04:05	LR
8	69	108	201	1	7	RPE	None	00:58	04:03	X
9	81	75	210	12.2	7	RT	None	01:13	04:25	Pelvic LN, abdominal LN, thoracic LN, pulm
10	67	80	197	1.25	7	RPE	None	00:57	04:00	LR

**Fig. 1:**
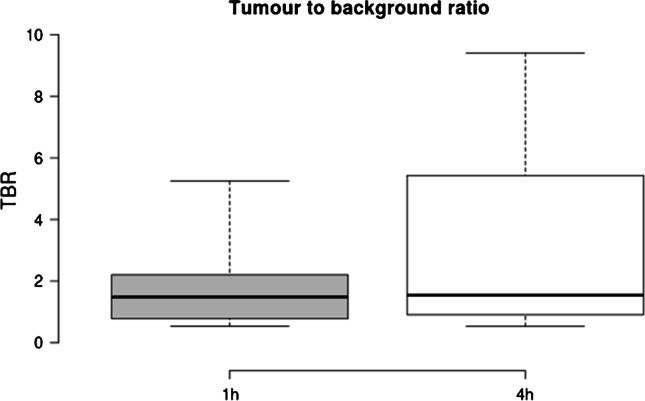
Shown are boxplots depicting tumour to background (TBR) ratio at 1 h (standard) acquisition, 4 h (late). Improved TBR is seen at 4 h p.i., and no significant reduction (*p* = 0.5) is seen with the patients undergoing a low dose protocol compared to standard acquisition. For all boxplots: the median is shown by the central line in bold, the 25th and 75th percentiles are shown by the box limits and whiskers extend to the minima and maxima

**Fig. 2:**
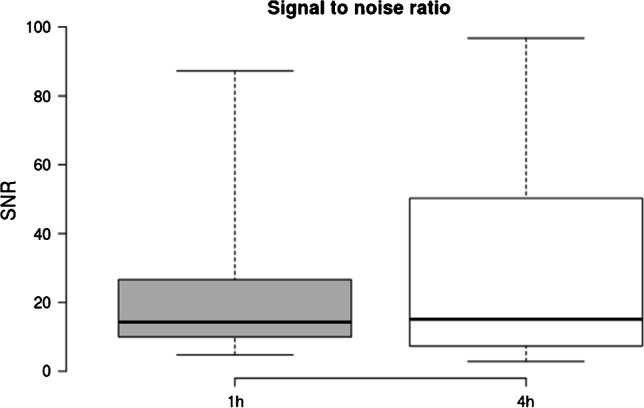
Signal to noise (SNR) ratio, defined as the reciprocal coefficient of variation for 1 h and 4 h acquisitions. Improved SNR is seen at later imaging

**Fig. 3:**
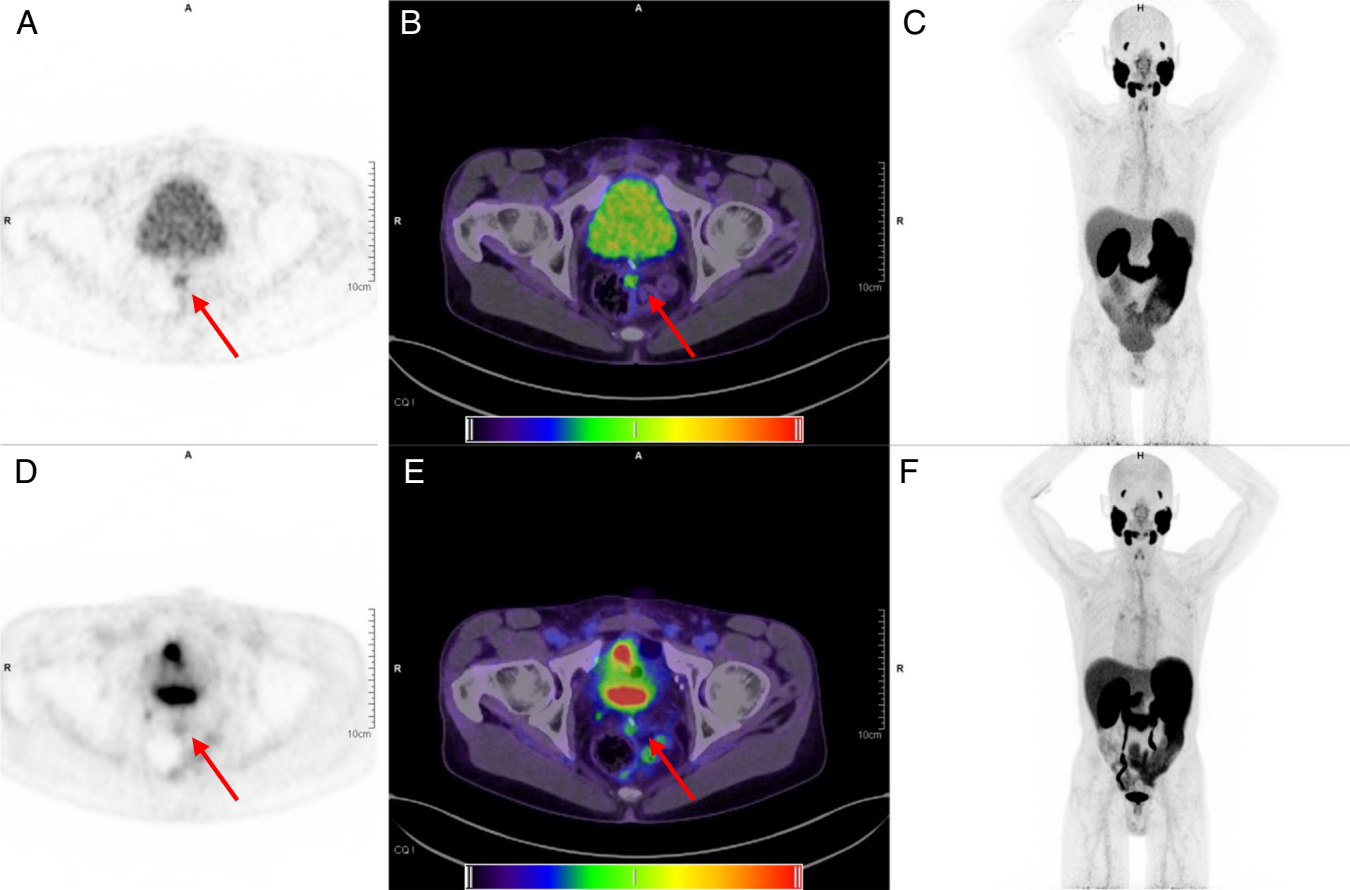
Illustrative images for an example patient (#3). Shown are images acquired at 4 h p.i. with a 16 min total acquisition time (top row, tiles **a**–**c**) and 1 h p.i. images with 6 min total acquisition time (bottom row, tiles **d**–**f**). Visual inspection of the two maximal intensity projections (**c** and **f**) demonstrates that only a modest reduction in image quality is seen at late imaging. The locally recurrent lesion (shown by red arrows) at the left mesorectal fascia is faintly visible at 1 h (PET **d** and fusion PET and CT, **e**) but more clearly discerned at 4 h (PET **a** and fusion PET and CT, **b**). For reference, the PET window was set to 0 and 6 SUV to best display the lesion

**Fig. 4:**
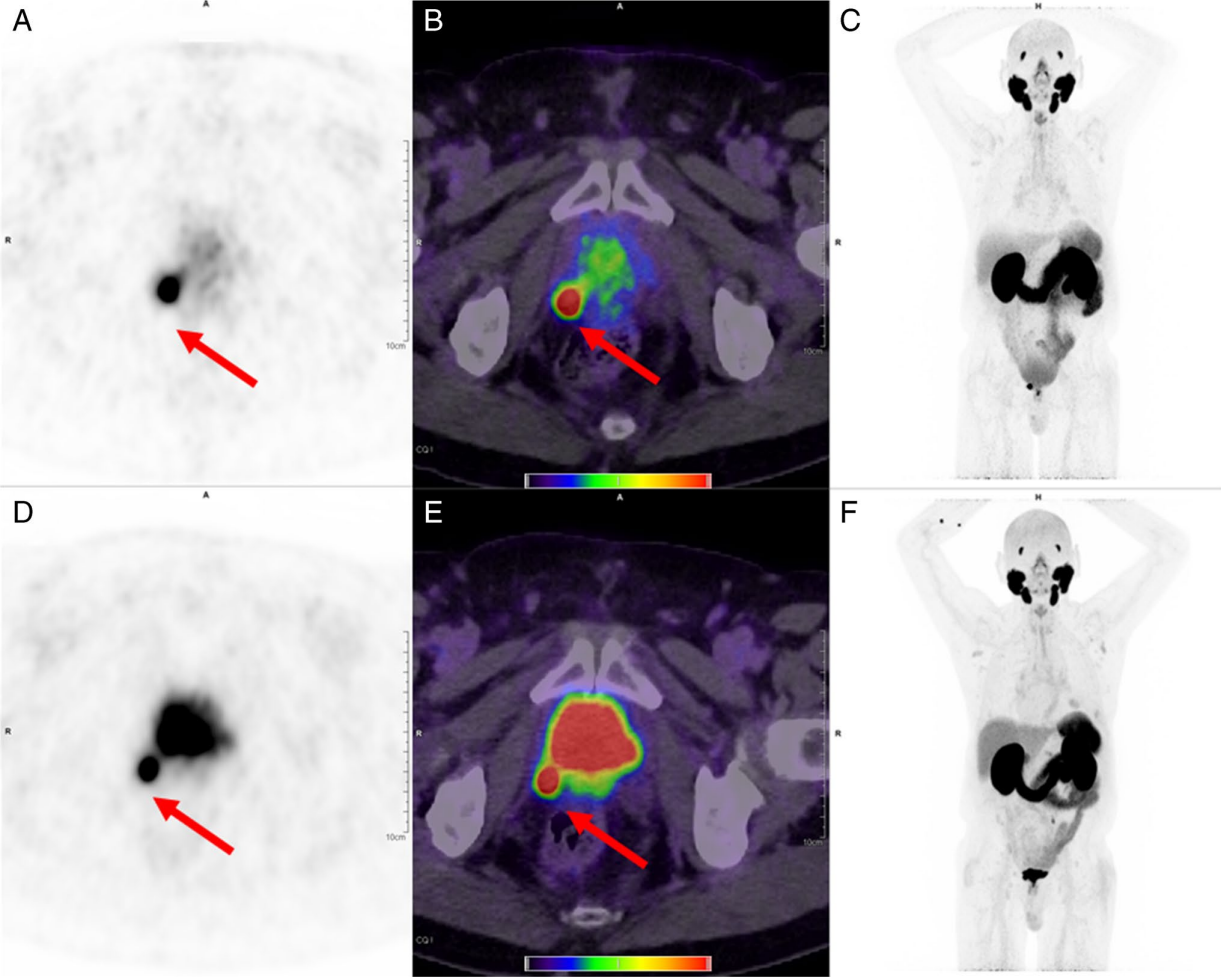
Illustrative images (patient #8). Shown are the 4 h images (top row) and 1 h images (bottom row). A locally recurrent lesion at the bladder wall (red arrow) is discernible in both the PET (tiles **a** and **d**) and the fusion PET and CT (tiles **b** and **e**), with barely perceptible visual difference in image quality between the two acquisitions. The combination of diuresis and later acquisition (at 4 h p.i.) results in better lesion demarcation from the bladder (MIP tiles **c** and **f**). Images are shown with PET window 0 to 10 SUV

Later acquisition was associated with higher lesion SUV_peak_ (mean 13.43 vs. 10.07, *p* = 0.05; median 8.46 vs. 6.51). Mean TBR was higher at 4 h compared to 1 h (3.41 vs 1.93, *p* < 0.001; median 1.55 vs. 1.48); data are shown in Fig. 1. SNR was improved at late acquisition (4 h p.i. 33.02 vs. 1 h p.i. 24.80, *p* = 0.062; median 15.1 vs 14.2), with borderline significance owing to the limited data in this case series. Data are shown in Fig. 2. Example patient images are shown in Fig. 3 and Fig. 4 and additional images in Supplementary Fig. 1 and 2.

## Discussion

This case series indicates that late imaging is feasible with [^68^ Ga]Ga-PSMA-11 without relevant loss of image quality, where the sensitivity gains in a LAFOV PET/CT system result in greater dynamic range, i.e. the greater sensitivity of the scanner allows radiotracers to be followed over more half-lives [1].

The pharmacokinetic behaviour of [^68^ Ga]Ga-PSMA-11 is well known, and the majority of tumour lesions show increasing radiotracer uptake over time [7]. It is therefore beneficial to observe the radiotracer over several half-lives, which can improve lesion visibility, background tissue clearance and TBR [10–14, 16, 22]. However, dual time point or later imaging is not universally accepted; additional or discretionary imaging can be challenging to integrate into the clinical routines of a busy nuclear medicine department and may require long acquisition times reducing scanner availability. With a mean applied activity for the patients in this report of 192 MBq, after 4 h, less than 17 MBq of the originally applied activity would be available following decay, and in reality even less would be available when considering the biological half-life [7]. With such low amounts of radiopharmaceutical activity remaining, the resultant imaging quality using previous-generation scanners was clinically unacceptable [23]. A standard acquisition using a SAFOV scanner recommended by the guidelines is 2 min/bp, which for a previous generation scanner in continuous bed motion could take up to 16 min (2 min/bp equivalent to 1.1 mm/s table speed [24]). By contrast, the LAFOV scanner is able to capture a 106 cm FOV in a single acquisition, enabling improved acquisition times: for example, a 16 min/bp acquisition, as we performed at 4 h, would be impracticably long when using a SAFOV system. Whereas later acquisition of images can be to the detriment of image quality using SAFOV systems, this is not the case for LAFOV systems. Moreover, these cases demonstrate the ability to achieve a high-quality image after nearly four half-lives had elapsed, i.e. where > 90% of the applied radiopharmaceutical has undergone decay. Although not the aim of this present study, this implies that as alternative to late acquisition, low dose protocols are feasible in LAFOV, for which further dedicated studies are required. Such low-dose protocols are of clinical interest since they might be a method to ameliorate supply bottlenecks for ^68^ Ga [25].

Despite nearly a decade of routine use, the optimal imaging protocol for [^68^ Ga]Ga-PSMA-11 PET/CT remains elusive. Given the improvement in lesion contrast (TBR) and SNR, rather than acquiring images at 1 h p.i., delayed acquisition might be preferable with new-generation LAFOV scanners. We note that later acquisition is recommended for other ^18^F-labelled PSMA-radiotracers, such as [^18^F]PSMA-1007 where 2 h p.i. is favoured [26] and where 2 h imaging may be valuable for [^18^F]DCFPyl [27].

Our short report is limited by the small patient numbers and represents our first experiences in this regard with this new scanner. Although readers were blinded to clinical details and scans were read in randomised order, recall bias cannot be excluded, although this case series does not seek to present a systematic evaluation but rather to demonstrate the feasibility of later image acquisition and imaging quality achievable on a state-of-the-art LAFOV system as a report of first clinical experiences with the first scanner of this type. Although further systematic analyses are required to confirm our findings, we hypothesise that when using LAFOV systems, later acquisitions could be favoured in certain conditions. Future studies should evaluate other imaging time points, such as 2 h or 3 h p.i.

These initial data demonstrate that, when using a LAFOV scanner, not only are late acquisitions feasible with total acquisition times not exceeding those of a routine scan, but that improvements in terms of TBR and SNR are possible.

## Conclusion

In this case series, late acquisition in tandem with a LAFOV PET/CT did not result in reduced imaging quality, however improved TBR and SNR at late imaging. High-quality imaging at a time point when nearly four half-lives for the radiotracer had elapsed was possible within clinically acceptable total acquisition times. These data show that later acquisition times for [^68^ Ga]Ga-PSMA-11 may be preferable when performed on LAFOV systems.

## Supplementary Information

Below is the link to the electronic supplementary material.

Supplementary file1 (DOCX 1.21 MB)
