# Supplementary material for: Feasibility of late acquisition [68Ga]Ga-PSMA-11 PET/CT using a long axial field-of-view PET/CT scanner for the diagnosis of recurrent prostate cancer—first clinical experiences
Source: Eur J Nucl Med Mol Imaging. 2021 Jun 21;48(13):4456–62. doi: 10.1007/s00259-021-05438-5 (PMC8566391; doi:10.1007/s00259-021-05438-5)
Supplement: Supplementary file 1 — Supplementary file1 (DOCX 1.21 MB) [file 259_2021_5438_MOESM1_ESM.docx]

**Supplementary images**


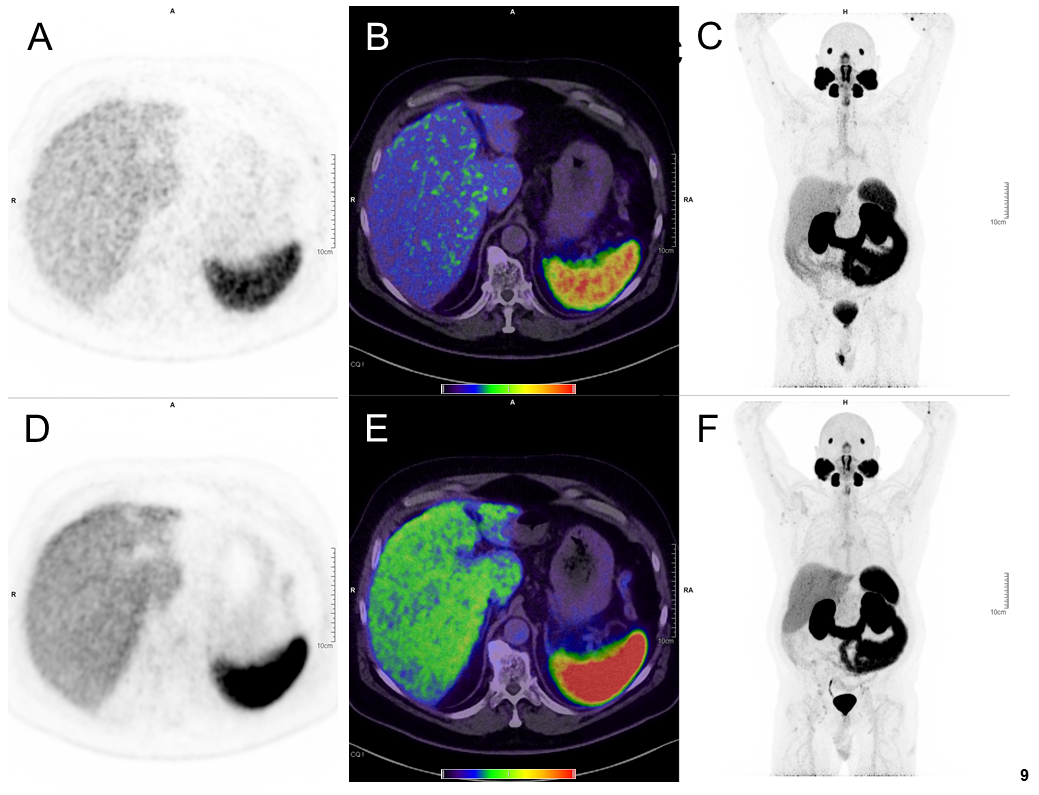


**Supplemental image 1**. Shown are the 4h images (top row) and 1h images (bottom row), with PET window 0 to 10 SUV. This patient shows no pathological lesions, but demonstrates how later acquisition of images results in improved tissue clearance (e.g. lower liver uptake at 4h).PET: A and D; fusion PET and CT: B and E; MIP tiles C and F.


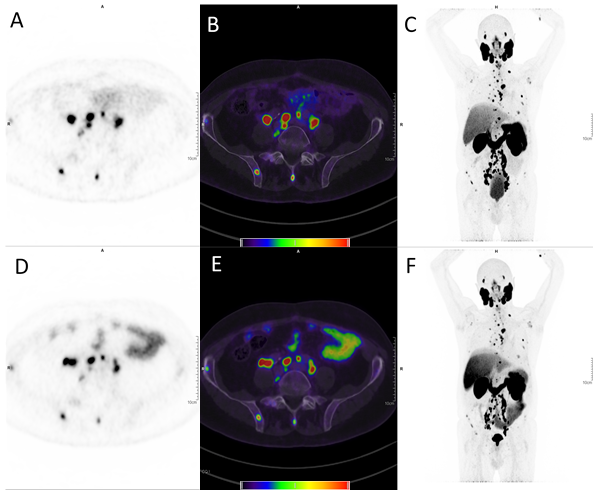


**Supplemental image 2**. Shown are the 4h images (top row) and 1h images (bottom row), with PET window 0 to 10 SUV. Multiple osseous and lymph node lesions are discernible in both the PET (tiles A and D) and the fusion PET and CT (tiles B and E where the CT is shown in the bone window), with barely perceptible visual difference in image quality between the two acquisitions. The later images show improved lesion uptake and lower background with improved soft tissue clearance (MIP tiles C and F).
